# The complete plastome and phylogeny of *Barnardia japonica* (Asparagaceae)

**DOI:** 10.1080/23802359.2020.1785350

**Published:** 2020-07-02

**Authors:** Li Chen, Ying Liu, Jianbo Chen, Na Lin, Maoqin Xia

**Affiliations:** a Jinhua Polytechnic, Jinhua, China; b Laboratory of Systematic & Evolutionary Botany and Biodiversity, College of Life Sciences, Zhejiang University, Hangzhou, China; c Lishui Hospital of Traditional Chinese Medicine, Lishui, China

**Keywords:** Asparagaceae, *Barnardia*, plastome, phylogeny

## Abstract

*Barnardia japonica* is a perennial herb in Asparagaceae, which has important ornamental and medicinal value. Here, we sequenced and characterized the plastome of *B. japonica* from Sichuan, China. The plastome length of *B. japonica* was 156,111 bp, including an 84,333 bp LSC, a 18,272 bp SSC and two 26,753 bp IRs. A total of 132 genes were detected, including 20 duplicated genes and 112 unique genes. Phylogenetic analysis based on 32 taxa revealed the monophyly of *B. japonica* and its sister relationship to *Albuca kirkii*. In addition, our results will provide valuable information for the phylogeny of Asparagaceae.

The genus *Barnardia* Lindley is perennial herb belonging to Asparagaceae, Scilloideae. It contains two species, *Barnardia japonica* (Thunb.) Schult. & Schult. f. and *Barnardia numidica* (Poir.) Speta, with the first one distributed in China, Japan, Korea, and Russia, while the other one in Northwest of Africa and Southwest of Europe. *Barnardia japonica* is an important ornamental and medicinal plant. Although Wang et al. (2018) have reported a complete plastome of *B. japonica* from Zhejiang, China, it could not fully represent the species because of its widespread distribution in both mountain and plain habitats, fits for wet to drought soil conditions and large morphological variation in filaments, leaf number and color of flower (Chang 2017). Moreover, the phylogeny of Asparagaceae is always a troublesome problem, and plastome sequences have been proved to be powerful to resolve phylogenetic relationships within and between families (Li et al. 2017; Liu et al. 2017). In this study, we sequenced another plastome of *B. japonica* from southwest China, and take advantage of published plastomes in Asparagaceae to compare the different characteristics of *B. japonica* plasome and also to provide a deep sight into the phylogeny of this family.

Fresh leaf samples were field-collected from Wenchuan County, Sichuan Province, China (31°23′25.38″N, 103°31′0.89″E), then dried with silica gel. Voucher herbarium specimen (*Pan Li PNLI20120098*) was deposited at the Herbarium of Zhejiang University (HZU). Total DNA was extracted from dried leaves using DNA Plantzol Reagent (Invitrogen, Carlsbad) following the manufacturer’s protocol, then sequenced using Illumina HiSeq 2500 platform (Illumina Inc., San Diego, CA). Raw data were assembled in NOVOPlasty 3.8.3 (Dierckxsens et al. 2017) with the published *B. japonica* (MH287351) as reference and its rbcL gene as seed. The contigs were arranged and annotated in Geneious 11.0.2 (http://www.geneious.com) with *Milla biflora* Cav. (NC_036000) as reference. The complete plastome circle was accomplished in OGDRAW (http://ogdraw.mpimp-golm.mpg.de/). Finally, the complete plastome sequence has been submitted to GenBank (MT319125). Thirty-one plastomes (28 species in Asparagaceae, 2 in Amaryllidaceae, 1 in Asphodelaceae and 1 in Iridaceae) were downloaded from GenBank. Phylogenetic analyses were implemented based on 86 CDS gene shared among all plastomes. The sequences were aligned using MAFFT in Geneious 11.0.2 (Katoh and Standley 2013). Maximum likelihood (ML) analysis was performed in RAxML-HPC v8.2.8 with 1000 bootstrap replicates (Miller et al. 2010). Bayesian analysis was constructed using MrBayes XSEDE 3.2.6 with two independent Markov Chain Monte Carlo chains for 10,000,000 generations and sampling every 1000 generations (Ronquist and Huelsenbeck 2003). The first 25% of calculated trees were discard as burn-in and the remaining trees were used to construct a consensus tree to estimate the posterior probability (PP). All phylogenetic analyses were conducted on CIPRES Science Gateway website (Miller et al. 2010).

The complete plastome of *Barnardia japonica* was 156,111 bp in length and presented a typical quadripartite structure, comprising a large single-copy (LSC, 84,333 bp) and a small single-copy (SSC, 18,272 bp) separated by a pair of inverted repeats (IR, 26,753 bp). The overall plastome GC content of was 37.7%. The plastome consisted a total of 132 genes, of which 112 were unique genes comprising 78 protein-coding genes (CDS), 30 tRNA genes, and 4 rRNA genes. These results were identical to previous published *B. japonica* (Wang et al. 2018).

The ML and BI phylogenetic trees showed congruent topologies with high supports (Figure 1). The monophyly of *B. japonica* was strongly supported based on CDS data (BS = 100, PP = 1; Figure 1). The closest genus of *B. japonica* was *Albuca* (BS = 100, PP = 1; Figure 1), rather than *Milla* suggested in Wang et al. (2018). In addition, the phylogenetic tree uncovered monophyly of seven subfamily in Asparagaceae, which provided an important reference of further phylogenetic study in this family.

**Figure 1.**
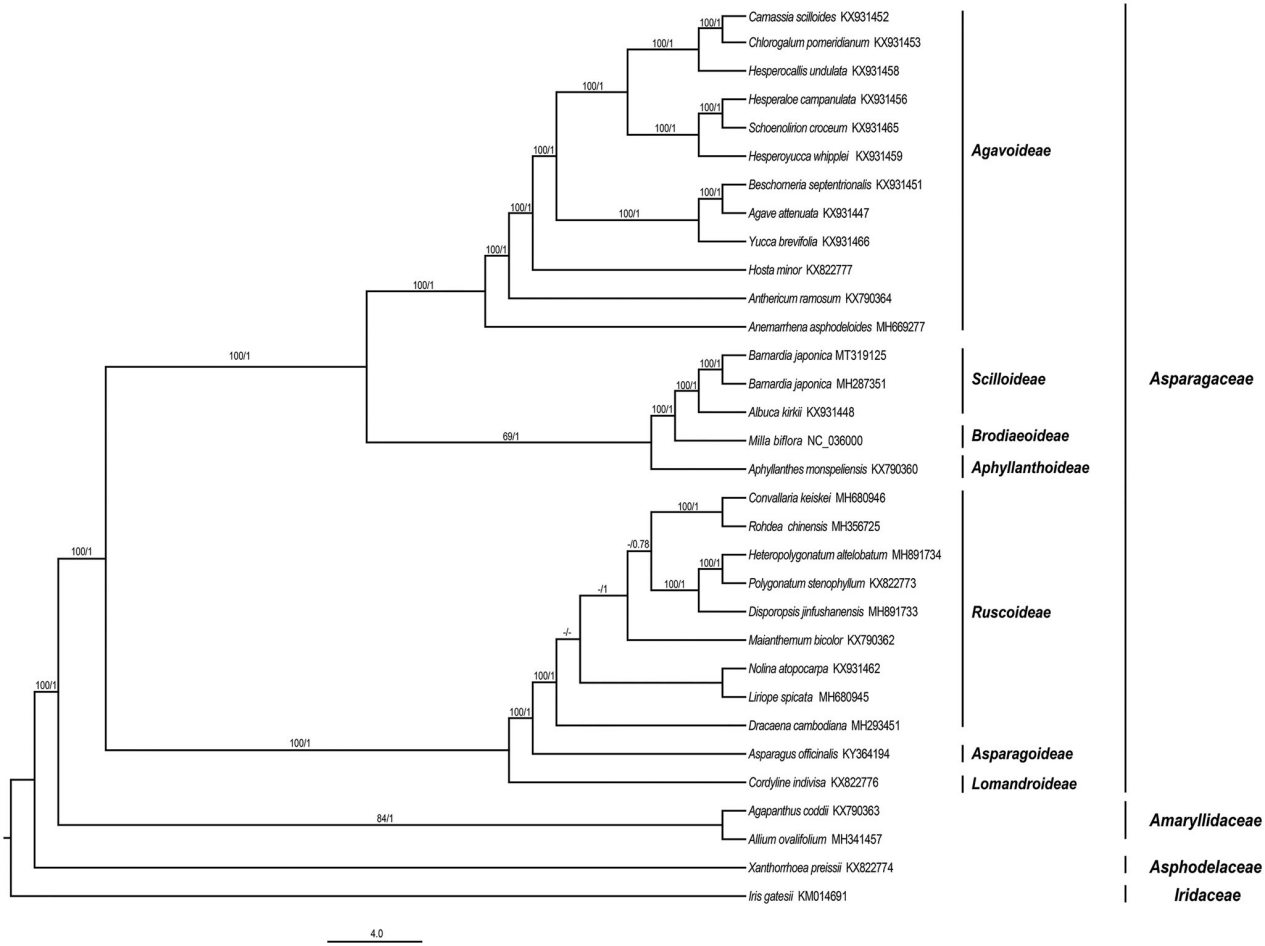
Phylogenetic relationships of Asparaceae inferred from Maximum likelihood (ML) and Bayesian inference (BI) methods based on 86 CDS genes. Numbers above the lines represent ML bootstrap values and BI posterior probability, - indicates support values less than 50%.

## Data Availability

The complete plastome of *Barnardia japonica* has been submitted to NCBI database (GenBank accession numbers: MT319125) at https://www.ncbi.nlm.nih.gov/nuccore/MT319125.
